# Forced Normalization and other neuro-psychiatric manifestations of epilepsy - Case series and A literature review

**DOI:** 10.1192/j.eurpsy.2022.438

**Published:** 2022-09-01

**Authors:** Y. Deol, M. Morales, M. San Gabriel, M. Du Boulay

**Affiliations:** Icahn School of Medicine at Mount Sinai, Psychiatry, Elmhurst, United States of America

**Keywords:** Alternative psychosis, forced normalization, psychosis of epilepsy, epilepsy

## Abstract

**Introduction:**

Psychosis of epilepsy has intrigued many neurologists and psychiatrists. We attempt to summarize the phenomenon, suggested diagnostic criteria and distinguishing features between different clinical entities linked with epilepsy.This case series is unique and rare as we include the case that meets full criteria of forced normalization.

**Objectives:**

1) To understand the concept and diagnostic criteria of Forced Normalization 2) To differentiate different psychiatric manifestations of epilepsy

**Methods:**

A total of 13 studies were reviewed using the key words from 1999 –2021 using different search engines- Google scholar, Pub-med, Elsevier, Dynamed.

**Results:**

Patients with epilepsy have an eightfold increased risk of psychosis (6). Forced Normalization has been described as the onset of psychotic or mood symptoms after the resolution or remission of >50% of seizures, evidenced by normal EEG. It was first described in 1950’s and has been extensively studied since 19^th^century. The age of onset can be 8 years to 71 years of age (mean - 28.3). The exact mechanism is still unknown. Different factors have been linked to this phenomenon like kindling, neurotransmitters etc.

**Conclusions:**

It is interesting to understand the antagonistic relationship between epilepsy and psychosis. Forced normalization is a rare entity because it is hard to diagnose due to possible overlap with other clinical entities like post-ictal or side effects of AED. The prognosis seems to be favorable depending on the trigger for the symptoms with better prognosis if the resolution of seizures was achieved AED. Mood disorders had worse prognosis than dissociation and psychosis.

**Disclosure:**

No significant relationships.

